# Supplementary material for: Differential activation of JAK-STAT signaling reveals functional compartmentalization in Drosophila blood progenitors
Source: eLife. 2021 Feb 17;10:e61409. doi: 10.7554/eLife.61409 (PMC7920551; doi:10.7554/eLife.61409)
Supplement: Transparent reporting form [file elife-61409-transrepform.pdf]

eLIFE
1st Floor
24 Hills Road
Cambridge CB2 1JP, UK
P 01223 855340
W elifesciences.org
T @elife

*eLife's*transparent reporting form

We encourage authors to provide detailed information *within their submission* to facilitate the interpretation and replication of experiments.Authors can upload supporting documentation to indicate the use of appropriate reporting guidelines for health-related research (see EQUATOR Network), life science research (see the BioSharing Information Resource), or the ARRIVE guidelines for reporting work involving animal research.Where applicable, authors should refer to any relevant reporting standards documents in this form.

If you have any questions, please consult our Journal Policiesand/or contact us:editorial@elifesciences.org.

Sample-size estimation

- You should state whether an appropriate sample size was computed when the study was being designed
- You should state the statistical method of sample size computation and any required assumptions
- If no explicit power analysis was used, you should describe how you decided what sample (replicate) size (number) to use

Please outline where this information can be found within the submission (e.g., sections or figure legends), or explain why this information doesn't apply to your submission:

Appropriate sample sizes were computed for every experiment, the sample size is denoted by (N) in the figures.
Sample (replicate) size (number) to use was based on relevant literature in this area.
Values plotted for every graph and the statistical method applied are mentioned in the source data.

Replicates

- You should report how often each experiment was performed
- You should include a definition of biological versus technical replication
- The data obtained should be provided and sufficient information should be provided to indicate the number of independent biological and/or technical replicates
- If you encountered any outliers, you should describe how these were handled
- Criteria for exclusion/inclusion of data should be clearly stated
- High-throughput sequence data should be uploaded before submission, with a private link for reviewers provided (these are available from both GEO and Array Express)

Please outline where this information can be found within the submission (e.g., sections or figure legends), or explain why this information doesn't apply to your submission:

Control and test genotypes were analyzed in parallel as and where applicable.
Each experiment was repeated independently at least three times. (N) Denotes biological replicate as each larva represents a distinct biological sample that is compared under similar conditions/ time points.
All data were included in analysis and graphs indicate the scatter or distribution of data points
Values that are plotted in the graph have been provided in the source data for respective figures.
Information regarding access to gene expression data is provided in the manuscript.

Statistical reporting

- Statistical analysis methods should be described and justified
- Raw data should be presented in figures whenever informative to do so (typically when N per group is less than 10)
- For each experiment, you should identify the statistical tests used, exact values of N, definitions of center, methods of multiple test correction, and dispersion and precision measures (e.g., mean, median, SD, SEM, confidence intervals; and, for the major substantive results, a measure of effect size (e.g., Pearson's r, Cohen's d)
- Report exact p-values wherever possible alongside the summary statistics and 95% confidence intervals. These should be reported for all key questions and not only when the p-value is less than 0.05.

Please outline where this information can be found within the submission (e.g., sections or figure legends), or explain why this information doesn't apply to your submission:

The statistical test applied in each case is mentioned in the figure legends and described in the source data.
Statistical significance is mentioned in the figures.

Exact p-values are mentioned in the source data for respective figures.
(For large datasets, or papers with a very large number of statistical tests, you may upload a single table file with tests, Ns, etc., with reference to sections in the manuscript.)

Group allocation

- Indicate how samples were allocated into experimental groups (in the case of clinical studies, please specify allocation to treatment method);if randomization was used, please also state if restricted randomization was applied
- Indicate if masking was used during group allocation, data collection and/or data analysis

Please outline where this information can be found within the submission (e.g., sections or figure legends), or explain why this information doesn't apply to your submission:

Not applicable

Additional data files ("source data")

- We encourage you to upload relevant additional data files, such asnumerical data that are represented as a graph in a figure, or as a summary table
- Where provided, these should be in the most useful format, and they can be uploaded as "Source data" files linked to a main figureor table
- Include model definition files including the full list of parameters used
- Include code used for data analysis (e.g., R, MatLab)
- Avoid statingthat data files are "available upon request"

Please indicate the figures or tables for which source data files have been provided:

Inventory of source data

Figure 1 source data 1: Contains numerical data plotted and statistical analysis related to Figure 1

Figure 1- figure supplement 1- source data 1: Contains numerical data plotted and statistical analysis related to Figure 1- figure supplement 1

Figure 1- figure supplement 2- source data 1: Contains numerical data plotted and statistical analysis related to Figure 1- figure supplement 2

Figure 3 source data 1: Contains numerical data plotted and statistical analysis related to Figure 3

Figure 4 source data 1: Contains numerical data plotted and statistical analysis related to Figure 4

Figure 4- figure supplement 1- source data 1: Contains numerical data plotted and statistical analysis related to Figure 4- figure supplement 1

Figure 4- figure supplement 2- source data 1: Contains numerical data plotted and statistical analysis related to Figure 4- figure supplement 2

Figure 5 source data 1: Contains numerical data plotted and statistical analysis related to Figure 5

Figure 5- figure supplement 1- source data 1: Contains numerical data plotted and statistical analysis related to Figure 5- figure supplement 1

Figure 5- figure supplement 2- source data 1: Contains numerical data plotted and statistical analysis related to Figure 5- figure supplement 2

Figure 6 source data 1: Contains numerical data plotted and statistical analysis related to Figure 6

Figure 7 source data 1: Contains numerical data plotted and statistical analysis related to Figure 7

Figure 7- figure supplement 1- source data 1: Contains numerical data plotted related to Figure 7- figure supplement 1
